# *Francisella tularensis* Transmission by Solid Organ Transplantation, 2017^1^

**DOI:** 10.3201/eid2504.181807

**Published:** 2019-04

**Authors:** Christina A. Nelson, Christian Murua, Jefferson M. Jones, Kelli Mohler, Ying Zhang, Landon Wiggins, Natalie A. Kwit, Laurel Respicio-Kingry, Luke C. Kingry, Jeannine M. Petersen, Jennifer Brown, Saima Aslam, Melissa Krafft, Shadaba Asad, Hikmat N. Dagher, John Ham, Luis H. Medina-Garcia, Kevin Burns, Walter E. Kelley, Alison F. Hinckley, Pallavi Annambhotla, Karen Carifo, Anthony Gonzalez, Elizabeth Helsel, Joseph Iser, Michael Johnson, Curtis L. Fritz, Sridhar V. Basavaraju, the Tularemia in Transplant Recipients Investigation Team

**Affiliations:** Centers for Disease Control and Prevention, Fort Collins, Colorado, USA (C.A. Nelson, N.A. Kwit, L. Respicio-Kingry, L.C. Kingry, J.M. Petersen, A.F. Hinckley);; Southern Nevada Health District, Las Vegas, Nevada, USA (C. Murua, Y. Zhang, K. Carifo, J. Iser, M. Johnson);; Centers for Disease Control and Prevention, Atlanta, Georgia, USA (J.M. Jones, P. Annambhotla, S.V. Basavaraju);; Phoenix Area Indian Health Service, Phoenix, Arizona, USA (K. Mohler, L. Wiggins, E. Helsel);; University of California Davis Medical Center, Sacramento, California, USA (J. Brown);; University of California, San Diego, California, USA (S. Aslam, M. Krafft);; University Medical Center of Southern Nevada, Las Vegas (S. Asad, J. Ham, L.H. Medina-Garcia);; Sunrise Hospital and Medical Center, Las Vegas (H.N. Dagher);; Nevada Donor Network, Las Vegas (K. Burns); American Red Cross, Salt Lake City, Utah, USA (W.E. Kelley);; University of Arizona College of Medicine, Tucson, Arizona, USA (W.E. Kelley);; Sacramento County Public Health Laboratory, Sacramento (A. Gonzalez); California Department of Public Health, Sacramento (C.L. Fritz)

**Keywords:** Tularemia, Francisella tularensis, transplant, tissue donors, transplantation, biological warfare, laboratory infection, prevention and control, bacteria, United States, bioterrorism and preparedness

## Abstract

In July 2017, fever and sepsis developed in 3 recipients of solid organs (1 heart and 2 kidneys) from a common donor in the United States; 1 of the kidney recipients died. Tularemia was suspected only after blood cultures from the surviving kidney recipient grew *Francisella* species. The organ donor, a middle-aged man from the southwestern United States, had been hospitalized for acute alcohol withdrawal syndrome, pneumonia, and multiorgan failure. *F. tularensis* subsp. *tularensis* (clade A2) was cultured from archived spleen tissue from the donor and blood from both kidney recipients. Whole-genome multilocus sequence typing indicated that the isolated strains were indistinguishable. The heart recipient remained seronegative with negative blood cultures but had been receiving antimicrobial drugs for a medical device infection before transplant. Two lagomorph carcasses collected near the donor’s residence were positive by PCR for *F. tularensis* subsp. *tularensis* (clade A2). This investigation documents *F. tularensis* transmission by solid organ transplantation.

Tularemia, also known as rabbit fever, is a zoonotic disease caused by the gram-negative bacterium *Francisella tularensis*. Natural transmission to humans occurs through a variety of routes, including tick and deerfly bites, direct handling of infected tissues, ingestion of contaminated water or tissues, or inhalation of infective materials (*1*). Tularemia occurs throughout the northern hemisphere in every US state except Hawaii. Each year in the United States, ≈120 cases are reported (*2*).

*F. tularensis* is a Tier 1 bioterrorism threat because of its low infective dose, ability to aerosolize, and history of development as a bioterrorism agent. Several countries have studied this organism or developed it as a bioweapon (*3*,*4*). *F. tularensis* has also caused laboratory-acquired infections (*5*); therefore, laboratory personnel must take specific precautionary measures when handling clinical isolates (*6*).

Clinical manifestations of *F. tularensis* infection depend on route of exposure. Ulceroglandular and glandular tularemia, characterized by fever and tender regional lymphadenopathy, are the most common forms and typically follow inoculation of the skin. Pneumonic tularemia, the most lethal form, results from inhalation of *F. tularensis* or hematogenous spread from local infection. Pneumonic tularemia typically produces fever, chest pain, shortness of breath, and variable radiographic findings. Oropharyngeal, oculoglandular, and typhoidal tularemia occur less frequently (*7*). The drugs typically recommended for treatment of tularemia are streptomycin, gentamicin, ciprofloxacin, or doxycycline (*4*).

In July 2017, the Centers for Disease Control and Prevention (CDC) was notified that blood cultures from 2 solid organ transplant recipients in different US states, both of whom had sepsis, yielded a small, gram-negative organism suspected to be *F. tularensis*. The 2 recipients shared a common organ donor, prompting a collaborative public health investigation to characterize the source of transmission.

## Patients

### Transplant Recipients

**Figure 1:**
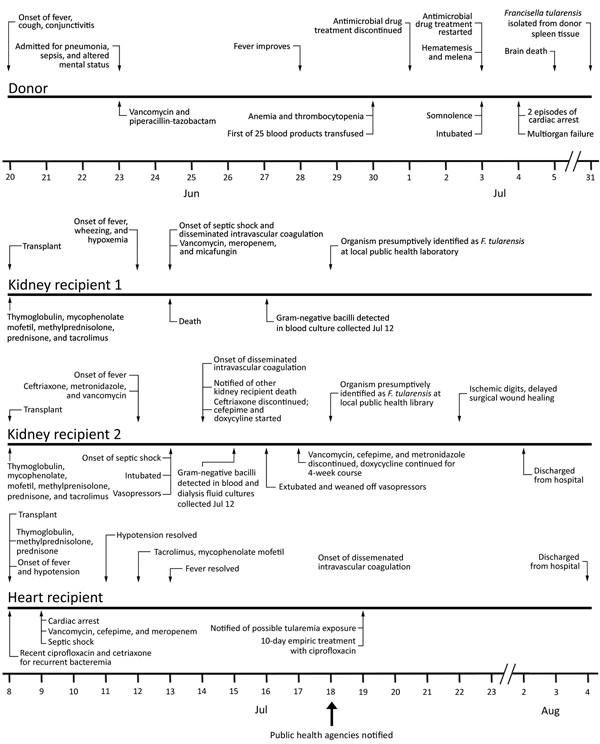
Clinical disease course for *Francisella tularensis*–infected organ donor and organ recipients, United States, 2017.

In July 2017, septic shock developed in 2 patients who had received a kidney and 1 patient who had received a heart from a common donor. The clinical course of disease, antimicrobial drug regimens, and immunosuppressive medications for these patients are summarized in Figure 1.

#### Kidney Recipient 1

The patient was a middle-aged man who had received a kidney transplant for IgA nephropathy and focal segmental glomerulosclerosis. Four days after transplantation, fever (39.4°C), hypoxemia, and wheezing developed. The next day, despite empiric treatment with vancomycin and meropenem, disseminated intravascular coagulation and septic shock developed, and the patient died. The organ procurement organization was alerted and immediately notified the physicians for the other 2 solid organ recipients.

Three days after the patient’s death, small gram-negative bacilli grew from a blood culture that had been collected 4 days after transplantation (Figure 1). Isolate characteristics subsequently suggested that it was a possible organism of bioterrorism (Select Agent); therefore, in accordance with American Society for Microbiology guidelines (https://www.asm.org/Articles/Policy/Laboratory-Response-Network-(LRN)-Sentinel-Level-C), the hospital laboratory discontinued identification procedures and referred the isolate to the local public health laboratory, a Laboratory Response Network (LRN) biological facility (*8*). Using growth characteristics, biochemical testing, and real-time PCR testing, the laboratory presumptively identified the organism as a *Francisella* species, notified CDC, and sent clinical samples to CDC for characterization.

#### Kidney Recipient 2

This patient was a woman in her 60s who had received a kidney transplant for diabetes mellitus–induced end-stage renal disease. Four days after transplantation, she experienced fever (39.4°C). Clinical samples were collected for culture, and treatment with vancomycin, ceftriaxone, and metronidazole was empirically initiated. She remained febrile; anemia and thrombocytopenia (49,000 platelets/mm^3^) developed, and she required mechanical ventilation and vasopressor support. Six days after transplantation, after the healthcare team was notified that the other kidney recipient had died of sepsis, doxycycline was added to the antimicrobial drug regimen. The patient eventually recovered.

Seven days after transplantation, gram-negative bacilli were isolated from blood and dialysate cultures (Figure 1). Gram-negative bacilli were also subsequently isolated from culture of samples from the peritoneal dialysis catheter tip and biliary fluid. Clinical samples were transferred to the local LRN biological facility and presumptively identified as a *Francisella* species (*8*). An isolate from the blood sample was sent to CDC for characterization.

#### Heart Recipient

The heart recipient was a middle-aged man with a history of nonischemic cardiomyopathy. Before transplantation, he had received ciprofloxacin and ceftriaxone for recurrent *Serratia marcescens* bacteremia related to an infected ventricular assist device. Ciprofloxacin was discontinued 1 day before transplantation and ceftriaxone the day of transplantation. Several hours after transplantation, fever (39.1°C), hypotension, and septic shock developed. He received vancomycin, meropenem, and cefepime, and fever resolved 5 days after transplantation. Results for cultures of blood collected during the septic episode, when the patient was receiving systemic antimicrobial drugs, were negative. Eleven days after transplantation, the clinical team was notified that the organ donor possibly had tularemia. The patient was empirically administered a 10-day course of oral ciprofloxacin and discharged home 27 days after transplantation.

### Organ Donor

**Figure 2:**
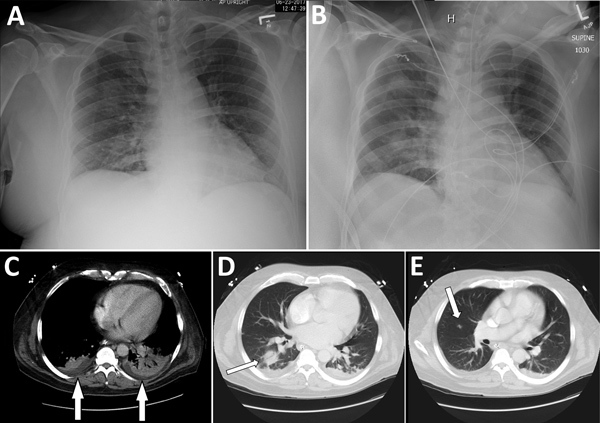
Radiographs (A, B) and computed tomography (C–E) images of chest of organ donor with *Francisella tularensis* infection, United States, 2017. Computed tomography images were taken after brain death. A) Anteroposterior view with patient in upright position, taken on day of admission; B) anteroposterior view with patient in supine position, taken on hospital day 10. C) Small bibasilar pleural effusions with adjacent subsegmental atelectasis versus pneumonia in the lower lobes (arrows); D) 3-cm round focus of pneumonia in the right lower lobe (arrow); E) 1-cm ill-defined nodule in the inferior right upper lobe (arrow).

In June 2017, a middle-aged alcoholic man was evaluated at an emergency department for obtundation, bloody emesis, fever (39.6°C), and respiratory distress. He had abruptly ceased alcohol intake 5 days before admission and experienced a nonproductive cough, nausea, headache, and conjunctivitis 3 days before admission. Chest radiographs revealed right upper and lower lobe infiltrates (Figure 2). Platelet count was low (108,000/mm^3^). Soon thereafter, the patient became hypoxic but did not require mechanical ventilation.

The patient was admitted for presumed aspiration pneumonia, sepsis, and alcohol withdrawal and administered piperacillin/tazobactam, vancomycin, and benzodiazepine. Five days later, his fever and mental status temporarily improved, but thrombocytopenia (range 1,000–26,000 platelets/mm^3^), bloody emesis, and melena progressively worsened. Over 7 days, he received 25 blood product transfusions. Chest radiographs demonstrated worsening interstitial opacities (Figure 2). During the next day, he experienced multiorgan failure and 2 cardiac arrests; 12 days after admission, he was declared brain dead.

Results of cultures of the donor’s blood (collected in blood culture bottles multiple times during hospitalization), urine, endotracheal aspirate, and bronchoalveolar lavage were all negative. Computed tomography of the chest performed after brain death revealed focal consolidation and a 1-cm nodule in the right perihilar region (Figure 2). Bronchoscopy indicated no abnormalities in the gross appearance of the lungs. An autopsy was not performed. Results of standard infectious disease testing for organ donor eligibility were negative (*9*). Both kidneys and the heart were procured for transplantation.

## Methods

We reviewed medical records of the organ donor and recipients and interviewed families and the 2 surviving patients about potential exposures to tularemia. In addition, we asked the organ donor’s family about the donor’s course of illness before hospitalization and illnesses of anyone who had been in close contact with him.

### Laboratory Testing

Recovered isolates were cultured at CDC on cysteine heart agar with 9% chocolatized sheep blood and confirmed as *F. tularensis* by direct fluorescent antibody (DFA) testing. DNA was extracted from organ donor and kidney recipient cultures and lagomorph bone marrow by using the QIAamp DNA MiniKit (QIAGEN, https://www.qiagen.com) and tested with real-time TaqMan PCR by using *F. tularensis* multitarget type A and type B assays, then A1 and A2 subtyping assays (*10*,*11*). Serum samples were tested for antibodies to *F. tularensis* by using the microagglutination assay (*6*); we considered a titer of >1:128 to be positive.

Pulsed-field gel electrophoresis (PFGE) typing of isolates was performed with the *PmeI* restriction enzyme (*12*) and clustered by using BioNumerics 6.64, Dice coefficient, and UPGMA (unweighted pair group method with arithmetic mean; Applied Maths, http://www.applied-maths.com). Whole-genome sequencing was performed by using Illumina V2 300 cycle reagents (*13*,*14*). The genome of each isolate was sequenced to an average depth of 311× coverage. To compare the isolated strains, we used whole-genome multilocus sequence typing (wgMLST) with the *F. tularensis* A2 strain WY96-3418 as a reference (GenBank accession no. NC_009257·1) (*15*). In brief, we mapped paired-end reads from each isolate to the WY96-3418 genome sequence and scanned 1,637 gene sequences (1,505,638 bp) representing 79% of the whole WY96-3418 genome (1,898,476 bp) for nucleotide differences by using CLC Genomics 10.0 (QIAGEN). Cluster analysis of alleles was performed in BioNumerics 7.5 (Applied Maths) by using categorical coefficient and UPGMA.

### Blood Donor Traceback

To determine whether infection may have been transmitted from an asymptomatic blood donor to the organ donor, we investigated the sources of all blood products transfused to the organ donor. Blood donors were asked about any potential exposures to tularemia and whether they had experienced febrile illness during the 2-week period after blood donation. Donors who reported potential exposures or febrile illness were tested for *F. tularensis* by serology.

### Environmental Assessment

We investigated the organ donor’s community—including the vicinity of the donor’s residence, neighboring homes, nearby fields, and a river swimming area—to identify animal carcasses or other evidence of recent rodent or lagomorph die-off. Presence of potential arthropod vectors such as deer flies was noted informally during the assessment; however, arthropods were not systematically collected as part of the investigation.

We also evaluated the residential water supply as a possible source of infection. Records from standard municipal water testing results were reviewed, and the source well for the donor’s residence was examined for evidence of compromise or animal contamination.

## Results

### Laboratory Tests

#### Culture, DFA, and PCR 

Testing of archived organ donor blood (collected in acid citrate dextrose and EDTA tubes 13 days after admission and stored at room temperature) and frozen spleen and lymph node tissues (collected 14 days after admission) produced the following results. Blood samples were negative for *F. tularensis* by PCR and culture (Table). *F. tularensis* was cultured from the spleen tissue; PCR genotyping of the isolate revealed that it was *F. tularensis* subsp. *tularensis* (clade A2). Results of DFA and direct PCR testing of spleen tissue were negative. No isolate was recovered from lymph node tissue. Isolates from kidney recipients 1 and 2 were confirmed as *F. tularensis* by DFA testing and determined to be *F. tularensis* subsp. *tularensis* (clade A2) by PCR genotyping.

**Table:** Culture and serology results for samples from *Francisella tularensis*–infected organ donor and organ recipients, United States, 2017

**Patient, outcome, samples tested**	**Results**
**Donor**	
** Blood**	Culture negative
** Endotracheal aspirate**	Culture negative
** Urine**	Culture negative
** Bilateral bronchial washes**	Culture negative
** Lymph node, after brain death**	Culture negative
** Spleen tissue, after brain death**	Culture positive for *Francisella tularensis*
** Serum, after brain death**	Serology positive (titer 1:128)*
**Kidney recipient 1, died**	
** Blood**	Culture positive for *F. tularensis*
** Cerebrospinal fluid, postmortem**	Culture positive for *F. tularensis*
** Kidney tissue, postmortem**	Culture positive for *F. tularensis*
** Bone marrow, postmortem**	Culture positive for *F. tularensis*
**Kidney recipient 2, discharged**	
** Blood**	Culture positive for *F. tularensis*
** Dialysate**	Culture positive for *F. tularensis*
** Biliary fluid**	Culture positive for *F. tularensis*
** Peritoneal dialysis catheter tip**	Culture positive for *F. tularensis*
**Heart recipient, discharged**	
** Blood**	Culture negative†
** Serum**	Serology negative

*Antibody titer may be artificially low because of the large volume of blood products and other fluids that the patient received. Presence of a mounted immune response, as evidenced by seropositivity, might have contributed to negative culture results in all specimens other than spleen tissue. **†Before transplantation, the patient received ciprofloxacin and ceftriaxone for bacteremia related to a colonized ventricular assist device. At the time of sample collection, the patient had received a final dose of ciprofloxacin the previous day and a final dose of ceftriaxone that morning. Subsequent blood cultures were collected while the patient was receiving antimicrobial drugs for presumed sepsis and were negative.**

#### Serologic Findings

Organ donor plasma from the archived EDTA blood specimen collected 13 days after admission was positive for antibodies to *F. tularensis* (titer 1:128). A serum sample from the heart recipient collected 11 days after transplant was negative for *F. tularensis* antibodies (titer <1:4).

#### Strain Types

**Figure 3:**
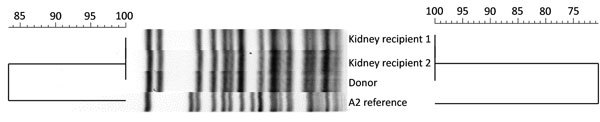
Pulsed-field gel electrophoresis (PFGE) and whole-genome multilocus sequence typing (wgMLST) comparisons of *Francisella tularensis *A2 strains. PFGE banding patterns and PFGE and wgMLST cluster analysis are shown for isolates from the organ donor and kidney recipients in relation to the *F. tularensis* A2 reference (strain WY96-3418). Dendrograms indicate percentage strain similarity for *PmeI *PFGE (left) and wgMLST (right).

Molecular typing of the *F. tularensis* A2 strains from the donor and both kidney recipients found all 3 to be indistinguishable from one another by 2 methods (Figure 3). *PmeI* PFGE banding patterns for the 3 strains were the same. Comparison of this banding pattern to a larger PFGE database of *PmeI* patterns for A2 strains from throughout the western United States (n = 30) demonstrated that the pattern was unique. Genome sequencing followed by wgMLST analysis revealed no nucleotide differences across 1.5 megabases of compared genome sequences between the 3 strains.

### Blood Donor Traceback Findings

Two blood suppliers provided the blood products transfused to the organ donor. Further blood donation by the donors who provided these products was temporarily deferred until the traceback and further serologic testing were completed. We identified and interviewed 43 of the blood donors. A review of blood supplier records confirmed that all blood donors met applicable regulations and standards at the time of donation. Telephone interviews of donors asked 9 questions specific to *F. tularensis* risk. One donor reported right-sided cervical lymphadenopathy; follow-up commercial laboratory testing of blood from this donor was negative for antibodies to *F. tularensis*.

### Environmental Assessment Findings

Interviews and medical record reviews of the organ recipients and their families revealed no known risk factors for tularemia. The organ donor resided on tribal lands in the southwestern United States. Family interviews revealed no known contact with sick animals, arthropod bites, household pets, or other noteworthy exposures. One family member reported observing groundhogs and rabbits near the donor’s residence but denied any apparent animal die-offs. The donor was unemployed and had not traveled outside the region for several months before his death.

No deer flies or other arthropod vectors were observed near the organ donor’s residence. Two lagomorph carcasses were found and collected ≈150 feet and 500 feet from the donor’s residence. No organs or soft tissue from the carcasses were available for testing; however, PCR testing of DNA from femur bone marrow followed by genotyping indicated that both lagomorphs were positive for *F. tularensis* subsp. *tularensis* (clade A2).

Maintenance records of the community water source, a municipal well and spring, did not indicate a breakdown of the chlorination process. Investigation of the well site revealed an intact well cover and functioning system with no evidence of animal entry.

## Discussion

We report human-to-human transmission of tularemia by solid organ transplantation. All 3 recipients of organs from a common donor with unrecognized tularemia became ill; 1 recipient died. *F. tularensis* infection in the donor and both kidney recipients was confirmed. Use of PFGE and wgMLST demonstrated that the *F. tularensis* isolates recovered from the donor and both kidney recipients were indiscernible from each other and distinct from other A2 strains, thereby corroborating transmission of *F. tularensis* A2 by solid organ transplant. Clinicians and organ procurement organizations evaluating potential organ donors who died of an unknown febrile illness should carefully assess risk factors for organ transplant–transmissible infectious diseases, including tularemia, and consider additional diagnostic testing if indicated (*6**,**16*).

The incubation period for naturally acquired tularemia is typically 3 to 5 days but can range from 1 to 21 days (*7*). For both kidney recipients, signs of infection developed 4 days after transplant, and the patients experienced rapidly progressive illness. Kidney recipient 2 might have survived because of empirically initiated ceftriaxone treatment. Ceftriaxone in vitro activity against *F. tularensis* has been demonstrated (*17*); however, in some cases ceftriaxone has been associated with treatment failure (*18*).

Although sepsis developed in the heart recipient several hours after transplantation, *F. tularensis* infection was not identified by culture or serology and would not be consistent with such a short incubation period. The episode of septic shock shortly after transplant might have been related to manipulation and removal of the infected medical device. It is possible that pretransplant ciprofloxacin for *Serratia marcescens* or posttransplant cefepime either prevented isolation of the organism or prevented *F. tularensis* infection altogether.

Human-to-human transmission of tularemia has been clearly documented just one time, in a medical examiner who accidentally cut her thumb during autopsy of a person who had died of tularemia (*19*). Another unconfirmed report from 1924 described a mother who contracted glandular tularemia after pricking her thumb while tending a tularemia ulcer on her son’s ear (*20*). 

Two subspecies of *F. tularensis* can result in human infection: subspecies *tularensis* (type A) and subspecies *holarctica* (type B) (*21*). PFGE analyses have further classified type A into 2 clades, A1 and A2, which differ in geographic distribution and case-fatality rate. A2 infections are known to occur only in the western United States, including the arid region from the Rocky Mountains west to the Sierra Nevada Mountains, matching the geographic location of the residence of the organ donor and the *F. tularensis*–positive lagomorph carcasses described in this report (*11*,*12*).

The organ donor might have been at increased risk for tularemia because of his residence on tribal lands. Native Americans are disproportionately represented among reported cases of tularemia; during 2001–2010, annual incidence among Native Americans was nearly 10 times higher than that of the general population (0.3 vs. 0.04 cases/100,000 persons) (*2*). Previous studies have estimated that 7%–17.5% of Native Americans residing in the United States and Canada have detectable *F. tularensis* antibodies (*22*–*26*). In addition, several outbreaks of tularemia among Native Americans have been reported, typically ascribed to tickborne infections (*26*–*29*).

Clinical and laboratory diagnoses of tularemia pose considerable challenges. Pneumonic tularemia, in particular, has myriad clinical forms and can mimic community-acquired pneumonia or other lung disorders (*1*,*7*). *F. tularensis* is not often isolated from blood cultures because it is fastidious and slow growing (*5*,*12*,*21*,*30*). For the organ donor reported in this article, blood culture sensitivity was probably further limited by neutralizing antibodies, given his positive tularemia serology results (*31*). Additional laboratory tests that may aid diagnosis include PCR and DFA, although these tests are not routinely performed unless disease is clinically suspected. Serology may aid diagnosis, although antibody responses are generally not detectable until 1–2 weeks after infection (*1*,*6*). Clinical laboratories must promptly report suspected *F. tularensis* infections to public health laboratories, according to LRN guidelines (*8*).

These findings are subject to limitations. First, the extent to which *F. tularensis* infection contributed to the donor’s clinical syndrome or death is unclear. Pulmonary findings may have resulted from aspiration pneumonia; gastrointestinal bleeding is not typically associated with tularemia but could have been precipitated by sepsis-related disseminated intravascular coagulation. In addition, we could not identify a specific exposure source for the donor. However, identification of 2 lagomorph carcasses near the donor’s home suggested a recent tularemia epizootic, and detection of *F. tularensis* A2 in the lagomorph carcasses confirmed presence of this organism near the donor’s residence.

Generally speaking, recipients’ risks for infection from any pathogen must be balanced with the growing shortage of organs (*32*). During the past 2 decades, several emerging or uncommon pathogens have been identified as potentially transmissible through solid organ transplantation (*33*,*34*), highlighting the challenges of identifying potentially transmissible pathogens in brain-dead organ donors (*32*). These unusual transplant-transmitted infections have prompted the addition of more questions on standard interviews administered to donors’ next of kin (*9*) and the performance of additional laboratory screening of donors at the discretion of organ procurement organizations. Real-time nucleic acid–based testing and other methods show promise for more rapidly and accurately identifying donor infections (*32*). All suspected donor-derived diseases should be reported to the Organ Procurement and Transplantation Network (https://optn.transplant.hrsa.gov), as was done in this investigation. 

*F. tularensis* continues to affect public health because of ongoing naturally acquired infections, potential to cause laboratory-acquired infections, status as a Tier 1 bioterrorism threat, and newly described human-to-human transmission. Clinicians should be aware of the possibility of *F. tularensis* infection in patients receiving organ transplants. When evaluating potential organ donors with febrile illnesses, clinicians should consider risk factors for tularemia such as recent contact with animal carcasses, arthropod bites, landscaping activities, residence in a rural area, and white or Native American race (*2*). If tularemia or other infectious diseases are suspected, this suspicion should be clearly conveyed to recipient transplant clinicians so that vigilant posttransplant clinical surveillance can be conducted and timely treatment initiated should a donor-derived infection occur.
